# Supplementary material for: Are carbon emissions trading and green financial instruments synergistic? -Comprehensive quantitative research based on content analysis
Source: PLoS One. 2024 Mar 7;19(3):e0298601. doi: 10.1371/journal.pone.0298601 (PMC10919601; doi:10.1371/journal.pone.0298601)
Supplement: S1 Data — (ZIP) [file pone.0298601.s001.zip › Data/地级市Z_F工作报告.zip!/╕╩╦α/╠∞╦«╩╨/└┤╫╘╣1⁄2╓┌║┼í╛┬φ┐╦╩2╛▌═°í┐_2014.docx]

各位代表:

现在,我代表市人民政府向大会报告工作,请予审议。并请各位政协委员和列席人员提出意见、建议。

2013年工作回顾

2013年,在省委、省政府和市委的坚强领导下,我们坚持以科学发展观为指导,认真贯彻党的十八大和省第十二次、市第六次党代会精神,积极应对经济转型和下行压力加大的严峻形势,努力克服暴洪等群发性地质灾害造成的不利影响,攻坚克难,真抓实干,完成了市六届人大二次会议确定的各项目标任务。预计实现生产总值480亿元,同比增长13%;大口径财政收入86.3亿元,增长19%,其中一般预算收入27.5亿元,增长19%;社会消费品零售总额200.3亿元,增长15%;城镇居民人均可支配收入17453元,增长15%;农民人均纯收入4482元,增长16%。

项目建设成效明显,发展基础不断夯实。完成固定资产投资541亿元,增长30%;100个市列重大项目完成投资250亿元。重点项目建设进展顺利,宝兰客运专线、天平铁路、十天高速、750千伏输变电、颖川河流域综合开发等项目加快建设,麦甘二级公路、天水西客站、宝天高速街亭温泉出口等工程启动实施。项目前期工作取得积极进展,天水机场迁建、张家川2×1000兆瓦电厂完成预可研,曲溪城乡供水工程完成可研及21个专项报告,天水至武都铁路、310国道秦州至武山段升级改造工程初设和天平高速公路、城区应急供水工程可研通过省级评审。

工业经济平稳运行,转型升级步伐加快。规模以上工业企业实现增加值103.5亿元,增长16%,其中“10强50户”企业实现增加值82亿元,增长23%。重点工业项目顺利实施,卷烟厂易地技改、长城电工电器产业园、华天科技产业园二期、星火产业园二期等项目加快建设。企业出城入园步伐加快,第一批入园企业进展顺利。实施承接产业转移项目173项,上海胜华西部电缆电器产业园、国家级电工产品综合检测试验研究基地等取得实质性进展。地质找矿行动继续开展,实施地质勘查项目27个。节能降耗和循环经济建设力度加大,实施节能与循环经济项目14个、重点污染减排项目60个。企业自主创新能力不断增强,新增省著名商标18件、省名牌产品13个、地理标志证明商标2件,星火机床有限公司荣获甘肃省政府质量奖。新发展非公经济市场主体4978户,非公经济增加值占生产总值比重达到38.9%,提高3.3个百分点。

特色产业不断壮大,农村经济稳步发展。实现农业增加值87亿元,增长7.5%。全年粮食总产量达到117.93万吨,连续十年增长。“48113”现代农业推进计划启动实施,果品、蔬菜、畜牧、劳务等优势产业加快发展,新建果品基地20.2万亩,新增蔬菜播种面积5万亩,新建标准化规模养殖场104个,输转务工人员68.55万人、劳务收入83.5亿元。八大示范园区建设进展良好,天水国家农业科技园区提质扩园有序推进,新增省、市级农业产业化重点龙头企业10家、农民专业合作社448家,新创建家庭农场36家。农业农村基础条件持续改善,新建农村户用沼气3000户,完成生态造林14.1万亩,建成防洪堤防151公里,新修梯田33.4万亩,治理水土流失面积333.73平方公里,解决了36.22万人安全饮水问题。“双联”行动深入推进,扶贫攻坚力度加大,实施整乡推进项目7个、整村推进项目75个,争取财政及“三西”专项扶贫资金2.77亿元,全年减贫19.88万人。抗灾救灾取得阶段性成效,灾区群众得到妥善安置,灾后重建有力有序推进,开工维修重建受灾群众住房3.83万户。

商贸旅游加速发展,第三产业活力增强。实现第三产业增加值197.5亿元,增长12%。商贸流通体系日益完善,天水商贸城、会展中心全面开工,甘谷物流园、武山洛门蔬菜批发市场、秦安小商品城建设进展良好。“

万村千乡”市场工程加快实施,建成农贸市场8个、农家店228个。旅游业发展势头良好,武山水帘洞、甘谷大像山晋升为国家4A级旅游景区,全年接待国内外游客1330万人次,实现旅游综合收入75.28亿元,分别增长28.14%和29.26%。金融服务体系不断健全,甘肃银行秦州区支行开业运营,众兴菌业、星火、风动等企业上市前期工作有序开展。新成立融资担保机构2家,总数达19家。发放“双联”惠农贷款4.66亿元、妇女小额担保贷款13.87亿元。

基础设施日益完善,城乡面貌逐步改观。新一轮城市总体规划修编工作进展顺利,渭河城区段防洪及环境治理工程基本完工,伏羲祭祀广场改扩建一期、清水轩辕湖公园全面竣工,赤峪路、成纪大道、孙家坪大桥、山水一号大桥等项目加快建设,金家庄公共文化活动中心、市体育中心等项目前期工作有序推进。五县县城和重点镇基础设施进一步完善,全市城镇化率达到33.39%。加大历史文化名城和传统村落保护力度,实施了贾家公馆、玉泉观等33处古建筑维修保护工程。实施农村环境连片整治示范项目52个,庄天二级公路清水段环境整治、秦安县百里生态文化长廊建设成效明显。

改革开放稳步推进,区域合作得到加强。行政管理体制改革步伐加快,取消下放行政审批事项66项。农村各项改革不断深化,新增土地流转面积14.8万亩,总流转面积达63万亩,完成集体林权流转3.15万亩。重点领域改革稳步推进,食品药品监管体制改革基本完成,市、县区广电网络整合工作全面完成,电子政务平台建设顺利推进,市公共资源交易平台建成运行。对外交流合作不断加强,成功举办了公祭伏羲大典暨第24届伏羲文化旅游节,我市荣获“全国最具魅力节庆城市”奖。招商引资成效明显,新签约招商引资项目205项,总投资549.21亿元,到位资金102.63亿元。对外贸易稳步增长,完成进出口总额3.6亿美元,增长16%。

社会事业全面进步,公共服务水平提升。教育基础条件不断改善,改造校舍33万平方米,消除中小学D级危房16.27万平方米,新建幼儿园79所,新建农村学校食堂7.72万平方米。科技创新步伐加快,实施科技项目308项,取得科技创新成果202项,98项达到国内先进以上水平,其中20项达到国际先进水平。文化体育事业加快发展,张家川马家塬、麦积牧马滩等遗址晋升为全国重点文物保护单位,麦积山石窟丝绸之路申遗通过联合国教科文组织的考察评估,广播电视“村村通”、“户户通”工程全面完成,《麦积圣歌》荣获第十四届“文华奖”优秀剧目奖,麦积山山地自行车邀请赛被评为全国体育旅游精品赛事,成功举办了市四运会。医疗卫生基础条件不断完善,市第四医院综合楼、市第三医院住院楼投入使用,建成村卫生室100个。新农合参合率达95.87%,23种农村重特大疾病纳入保障范围,医疗费用补偿比例达70%,我市被确定为国家中医药养生保健旅游创新区。人口自然增长率控制在7‰以内。全国第三次经济普查顺利开展。审计、地震、人防、气象、外事侨务、地方志、档案、老龄、妇女儿童、残疾人以及国防动员、双拥共建等工作取得新进步。

人民生活持续改善,社会大局和谐稳定。省列10项26件、市列12件民生实事全面落实。开工建设保障性住房1.2万套,主体竣工8681套,发放廉租住房租赁补贴2.15万户4978万元,改造农村危房1.12万户。新增城镇就业6.16万人,城镇登记失业率3.06%。城乡居民社会养老保险参保率达95%、待遇发放率达100%。社会救助力度不断加大,发放城市低保金2.78亿元、农村低保金4.93亿元、五保户供养费4639万元、城乡医疗救助金9755.6万元、困难群众临时救助金5948万元,下拨各类救灾资金1.21亿元。麦积全国综合养老示范基地项目顺利实施。平安天水建设逐步深化,矛盾纠纷排查调处力度加大,一些重点疑难信访案件得到解决。“六

五”普法深入开展,民主法制和法治政府建设不断加强,办理人大代表建议191件、政协委员提案244件,办结率均为100%。认真贯彻落实中央和省、市委关于改进工作作风、密切联系群众的一系列政策规定,政府自身建设全面加强。社会治安秩序持续好转,安全生产形势总体平稳,市场物价和食品供应保持基本稳定。民族宗教工作进一步加强,成功举办了张家川县成立60周年县庆活动。

各位代表,2013年,我们经受了百年不遇暴洪灾害的严峻考验,我们实施了一系列改革开放的重要举措,我们取得了经济社会发展的明显成绩。这是省委、省政府和市委科学决策、正确领导的结果,是市人大、市政协及社会各界监督、支持的结果,是全市人民众志成城、奋力拼搏的结果。为此,我代表市人民政府,向全市各族人民、各民主党派、各人民团体、工商联和社会各界人士,向驻市人民解放军、武警消防官兵和驻市单位,表示崇高的敬意和衷心的感谢!

各位代表,在肯定成绩的同时,我们也清醒地看到,全市经济社会发展及政府工作中还存在一些困难和问题,主要是:发展速度较慢,经济总量偏小,整体实力不强,实现转型跨越的压力很大;产业规模不大,发展水平较低,促进产业结构优化升级的难度较大;城乡二元结构矛盾突出,经济社会发展活力不足,全面深化改革任务艰巨;城市建设相对滞后,加快城镇化进程需要付出艰辛努力;社会事业发展不平衡,公共服务水平有待提升;社会保障体系还不完善,城乡居民收入水平低,社会就业压力大,灾后重建任务重;社会治理仍需加强,维护稳定的任务依然繁重;发展环境不够宽松,政府职能还需加快转变,工作作风亟待进一步改进。对于这些问题,我们将采取有力措施,逐步加以解决。

2014年奋斗目标和主要任务

2014年是我市改革发展进入重要历史阶段的关键一年。尽管我市经济社会发展存在着不容忽视的矛盾和问题,但更面临着一系列重大机遇和利好政策。党的十八届三中全会对全面深化改革作出了战略部署,有利于我们破除发展中的体制机制障碍,增强加快发展的动力活力;国家深入实施西部大开发、支持甘肃经济社会发展,有利于我们争取更多的项目、资金和政策支持,实现借力发展;国家建设关中—天水经济区和丝绸之路经济带,省上提出打造丝绸之路甘肃黄金段,有利于我们进一步扩大开放开发,在更宽领域、更高层次实现区域交流与合作;国家推进华夏文明传承创新区建设,有利于我们充分发挥历史文化和旅游资源优势,全力打造历史文化名城和全球华人寻根祭祖圣地;最近召开的全国城镇化工作会议,在国家规划的全国“两横三纵”城市化战略格局中,将我市列为“一横一纵”城市发展群的节点城市,为我市城镇化发展带来了新的历史机遇;我市被列入国家六盘山片区扶贫开发规划,有利于我们加大扶贫开发力度,促进城乡统筹发展;特别是我市已经打造的国家级经济技术开发区、国家农业科技园区等国家级金字招牌,提升了聚集生产要素的能力,为我们加快改革发展搭建了战略平台。机遇叠加,时不我待,只要我们审时度势,奋发进取,把更多的精力放在凝聚正能量、抢抓大机遇、推动大发展上,就一定能够推动全市经济社会转型跨越发展。

2014年政府工作的指导思想是:深入贯彻落实中央和省委、省政府的决策部署,围绕全面建成小康社会总目标和“十二五”经济社会发展规划,坚持市委、市政府确定的“关天经济区引领、国家级园区支撑、现代化城市带动、县区组团式发展”的基本思路,以加快发展为主题,以改革开放为动力,以“3341”项目工程为载体,稳中求进,好中求快,改革创新,务求实效,全力推进区域性现代化大城市建设,为建设富裕文明、幸福美好的新天水而努力奋斗。

经济社会发展的主要预期目标是:

生产总值增长11.5%,固定资产投资增长25%,规模以上工业增加值增长15%,大口径财政收入增长15%,社会消费品零售总额增长14%,城镇居民人均可支配收入增长13%,农民人均纯收入增长15%,居民消费价格指数控制在103.5%左右,城镇登记失业率控制在4%以内,人口自然增长率控制在7.3‰以内,万元生产总值综合能耗下降3.2%。

围绕上述目标,市政府将重点抓好以下九个方面的工作:

一、大力发展现代农业,拓宽农民增收致富新渠道

高度重视“三农”工作,全面实施“48113”现代农业推进计划和“1461”扶贫攻坚工程,促进农业增效、农民增收、农村发展。

做强四大主导产业。以持续打造果品、蔬菜、畜牧、劳务四个百亿元主导产业为目标,按照标准化扩基地、规范化保质量、品牌化促营销的要求,加快建设果品产业“五大特色基地”、蔬菜产业“一区两园三基地”和畜牧产业“四大养殖基地”,当年新建果品基地15万亩,新增蔬菜面积5万亩,新建扩建规模化养殖场(小区)95个,输转农村富余劳动力68.5万人,创劳务收入87.5亿元。重视加强马铃薯、中药材、花卉等特色产业发展。

建设八大示范园区。高起点、高标准加快建设市上确定的花牛苹果产业园、武山蔬菜科技园等8个现代农业示范园区,重点在基础设施建设、科技示范推广、产业聚集发展和服务体系建设等方面取得新的突破,充分发挥园区建设在全市农业产业开发中的示范引领作用。继续抓好天水国家农业科技园区的提质扩园工作,做好做优航天育种产业。抓好100个农业科学发展示范点建设,通过典型引路推动农业农村工作上水平。

实施十个百万工程。继续实施科技增粮、土地流转、优质梯田、安全饮水和林业生态建设等10个百万工程,重点在加强农业基础建设、改善农业生产条件、提升农业开发水平上积蓄实力、增强后劲。以确保粮食安全为前提,当年粮食面积稳定在470万亩左右,力争总产达到118万吨以上;农村土地流转总面积达到72万亩,流转率达到13%;新修梯田34万亩,新建堤防工程120公里,治理水土流失面积240平方公里,完成生态造林15万亩。

培育百强龙头企业。坚持规模化经营、集约化生产、产业化带动,加快培育新型农业经营主体,打造一批生产规模大、加工水平高、带动能力强的龙头企业。当年重点扶持发展龙头企业40家,新注册农民专业合作社210家,新发展家庭农场80家。

抓好扶贫攻坚和灾后重建工作。按照省上实施“1236”扶贫攻坚行动的总体要求,认真实施我市“1461”扶贫攻坚工程,深入推进“双联”行动,坚持整乡整村整流域整片带开发扶贫,集中力量打好扶贫攻坚战,实现27.72万贫困人口脱贫。高度重视灾后重建工作,整合灾后重建、农村危房改造、易地搬迁、财政“一事一议”、扶贫开发等项目资金,科学规划,加快进度,全面完成农村灾后重建工作任务。

二、壮大工业经济实力,力促工业转型升级迈上新台阶

按照“突出一个振兴、建设一区多园、发展六大集群、壮大县域工业”的思路,进一步强化工业主导地位,坚定不移地实施“工业强市”战略,促进工业经济转型升级。

全力推动工业企业提质扩量。继续深入实施工业“10强50户”工程和“12341”行动计划,着力抓好促进工业企业发展提质扩量工程:倾力打造5户以上年销售收入上50亿元的产业化企业,培育提升10户以上年销售收入上10亿元的骨干企业,支持发展30户以上年销售收入上亿元的成长性企业。加快天水国家经济技术开发区增容扩区,让各县区的工业园区共同享受开发区的优惠政策,尽快启动开发区在甘谷武山渭河川道区域的扩区工作。加快工业企业出城入园进度,做好第二批企业的出城入园工作。高度重视县域工业经济发展,办好工业集中区,使工业在提

升县域经济实力中发挥更加重要作用。

着力培育工业优势主导产业。围绕打造全国先进制造业基地,以华天、星火、长城电工等企业为龙头,做优做强特色优势产业,积极发展新兴高端产业,扶持壮大优势骨干企业,努力把全市有一定基础和发展潜能的机械制造、电工电器、电子信息、医药食品、建筑建材、能源化工等六大工业集群打造成百亿元的产业集群。深化企业改革,推进企业上市,重点做好众兴菌业及星火、风动、锻压、海林等企业的股权优化和上市工作。加大地质找矿工作力度,坚持依法管矿办矿,把地质资源优势科学有序地转化为经济优势。

高度重视工业项目建设。认真抓好目前有一定基础的胜华电缆产业园、蓝宝石加工、娃哈哈三期、武山祁连水泥技改扩建、瑞霖制药艾福康生产线、挪威小型掘进机、大飞机舱门、国家级电工产品综合检测实验研究基地、防霜机等一批工业企业项目的开工建设,加快天水卷烟厂易地技改、长城电工电器产业园、华天科技产业园二期、张家川太极集团阿胶生产线等项目建设进度,不断引进实施新的工业项目,进一步增强工业经济后续发展能力。

促进企业技术进步与创新。深入实施质量振兴和商标战略,发挥企业技术创新主体作用,支持重点企业加快装备升级改造,引导企业建设技术(工程)中心、重点实验室等创新平台,推动信息化和工业化深度融合。争创省级以上企业技术中心2家、省级和国家级技术创新示范企业2家,省级以上名牌产品8件、省级知名品牌示范区1个,中国驰名商标2件、甘肃省著名商标10件。

支持小微企业和非公经济发展。坚持权利平等、机会平等、规则平等,消除各种隐性壁垒,在融资、创业、技术、人才、培训、信息等公共服务方面创造公平竞争的环境,鼓励有条件的私营企业建立现代企业制度,积极发展混合所有制经济,支持民营企业进入更多领域从事经营开发。

三、坚持实施项目带动战略,实现重大项目建设新突破

始终把项目建设作为加快发展、实现跨越的重大战略举措,积极争取项目,扎实推进项目,超前谋划项目,切实把项目工作抓得更紧、更实、更好。

加大力度实施“3341”项目工程。围绕打造区域中心城市和交通运输枢纽城市,切实重视和加强交通、能源、电讯等基础设施建设。加快宝兰客运专线、天平铁路、十天高速、宝天高速街亭温泉出口、天水西客站、麦积客运站、麦甘二级公路、张家川县城至恭门火车站二级公路、洛礼二级公路建设进度,推进天水机场迁建、天水至武都铁路、天水高铁综合客运枢纽站、天平高速公路、310国道秦州至武山段升级改造、秦州至三阳川隧道等项目的基础工作。围绕提高水资源配置能力和保障城市发展用水,争取实施城区应急供水、曲溪城乡供水等工程,加快秦安小湾河等水库建设。围绕省上建设陇东能源化工基地,抓好张家川煤电冶一体化、天源科创张家川风电场一期、市区集中供热管网改造、各县区通天然气等项目,争取实施清水2×1000兆瓦火电项目,建成750千伏输变电工程。加强信息基础设施建设,实施“智慧天水”信息化等项目,年内开通4G通信网络平台,实现宽带网络城乡全覆盖。

精心谋划和论证储备后续发展项目。充分发挥比较优势,突出特色产业升级、城市基础设施建设、矿产资源开发、节能环保、文化旅游、信息消费、农产品和轻工产品外销等重点领域,谋划、论证、储备一批符合产业发展政策、事关我市长远发展的大项目、好项目,把发展机遇和政策优势转化为实实在在的项目。抓住国际国内产业分工调整机遇,以装备制造业、能源原材料产业、高新技术产业、现代服务业为重点,积极承接发达地区和大企业产业转移。

继续优化项目建设投资发展环境。完善领导干部包抓重大项目责任制和县区、部门目标责任制,大力

推行一站式服务、限时办结制,做好项目征地拆迁、基础设施配套等保障服务工作,完善投融资机制,优化投资环境,推动项目顺利实施。高度重视项目库建设,切实加强“3341”项目的动态管理。严格项目建设和资金监管,确保项目建设的质量和效益。

四、加快建设美丽天水,构建城镇化发展新格局

围绕破解城乡二元结构,加快城乡一体化进程,加强城镇基础设施建设,提升生态文明水平,推动区域性现代化大城市建设。

高度重视城市扩容和旧城改造工作。按照“东西延伸、南北扩展、相向对接、旧城改造”的总体思路,以316国道二级路改造为契机,启动西十里至藉口镇的城市干道建设项目,做好城区到皂郊二级路改造后的绿化亮化工作,以此带动相关区域项目的实施,促进城区向西延伸发展;通过对马跑泉至甘泉道路按城市道路绿化亮化、实施潘集寨到街子温泉二级路项目、社棠工业园区商务区建设以及相关项目的实施,带动城区向社棠、颖川河、东柯河流域的东部延伸;通过实施成纪大道建设、成纪新城建设、藉河生态治理二期工程等项目,实现两区在藉河以北的相向对接;通过对南北两山路网建设、通水工程以及相关项目的实施,带动促进城市空间向南北两山扩展;按照规划重点抓好西十里、岷山厂、东方红村、枣园村、客运专线火车站等片区的改造,做好土地熟化、城中村改造、棚户区改造、商业地产开发和工业企业出城入园等工作,有序推进原有城区的改造修复工作。

集中力量抓好城市重点项目建设。推进城区畅通工程建设,全面完成赤峪路市政道路、藉河孙家坪大桥、藉河山水一号大桥、渭河社棠大桥等项目,加快成纪大道、渭河峡口大桥、麦积新城路网、城区道路出口改造等项目建设,启动藉河双桥大桥、藉河南路西延、双桥南路拓宽、麦积区府路西延、马跑泉中心大道东延等工程,完善城区交通网络。加快市商务中心、岷山厂片区市政路网、城区排涝工程和秦州区集中供热等公用设施建设,统筹抓好社区市场、公共停车场、公厕、垃圾污水处理和水电暖气等市政设施建设,实施小巷道硬化亮化工程,加快城市建设步伐,进一步完善城市综合服务功能。

稳步推进城镇化和城乡一体化进程。坚持走新型城镇化道路,推进中心市区、县城与小城镇的协调发展,促进产业与城镇的融合发展,提高城镇容纳人口、吸纳就业和综合承载能力。加快城乡一体化发展,重点打造天定高速公路、庄天二级公路、310国道天水段沿线三条城乡发展一体化示范长廊。有序放开县城和建制镇落户限制,推进农业转移人口市民化,加快城乡基本公共服务均等化进程。深入开展“整治城乡环境、推进三城联创、建设美丽天水”活动,健全城镇管理长效机制,提高市民文明素质,提升城镇文明水平。

重视加强城市规划和规划执法工作。修编完善城市总体规划,抓紧做好二十里铺中央商务区、三阳川新城功能区、麦积颖川河东柯河新城区等城市新区的规划,完善藉河南路、岷山厂等片区更新改造和藉河生态治理二期工程、南北两山生态环境保护与公共空间利用、成纪新城、秦州城区西扩等专项规划。按照分级负责的原则,进一步加强村镇规划工作。加强规划执法工作,强化城镇规划的刚性约束力。

继续做好历史文化名城保护工作。完成《天水市历史文化名城保护规划》、《天水伏羲城修建性详细规划》等修编工作,加快历史文化街区、古民居的修缮保护建设,启动实施西关片区综合保护与复兴项目,做好城区古树名木保护复壮工作。加强历史文化名镇名村和传统村落保护工作,筛选推荐一批列入市级以上保护名录,促进民风民俗文化的传承发展。

全面推进生态文明建设。以创建“国家园林城市”为目标,加强城市园林绿化和景观工程建设,加快实施马跑泉广场、王家磨滨河道路

和山水新城道路景观、渭河北子堤生态公园项目工程,开工建设马跑泉公园扩建、秦州北山公园、湿地公园、秦州文化园和成纪新城北部生态公园、山地体育公园等工程。大力发展循环经济,积极推广节水、节能、环保新技术新工艺,抓好工业节能降耗和循环经济项目的实施。深入实施蓝天、碧水、安全、宁静、生态五大环保工程,加大重点污染源、重点流域专项治理,全面完成节能减排目标任务。

五、提速发展商贸旅游业,培育第三产业增长新亮点

坚持把发展以商贸旅游为主的第三产业作为调结构、保增长的重点,力促第三产业发展提速、比重提高。

重视加强商贸物流基础设施建设。坚持实施“商贸活市”战略,以建设陇东南现代商贸物流中心城市为目标,加强商贸流通项目建设,加快天水会展中心、天水商贸城、甘谷物流园、秦安小商品城、清水兴盛物流园、武山洛门蔬菜批发市场、天水城市蔬菜仓储分拣中心等项目的建设进度。启动天水汽车博览城、麦积东柯物流园、秦州藉口物流园、瀛池蔬菜批发市场、天水肉菜流通追溯体系等项目的规划建设。实施“万村千乡”市场工程,实现县区商品配送中心全覆盖。

发挥优势做大旅游产业。完善旅游业大景区建设发展规划,加强旅游基础和配套设施建设,改革旅游管理体制,巩固提升麦积山、伏羲庙、大地湾等重点旅游景区质量。加强文化旅游项目建设,创新宣传营销方式,强化旅游市场监管,提升旅游服务质量,打造丝绸之路经济带重点旅游城市。大力发展乡村旅游,规范农家乐管理,引导旅游企业晋等升级,促进旅游业持续快速健康发展,旅游接待人次和综合收入同步增长28%。

支持发展现代服务业。完善现代商贸流通网络,鼓励发展大型超市、连锁经营、物流配送、电子商务等新兴服务业,加快交通运输、邮政通信和金融业发展,培育科技、法律、信息服务及休闲娱乐等新型产业,打造重要商品市场监测调控平台。支持农超对接、家政服务、再生资源回收、老字号企业快速发展,促进便民消费进社区、便民服务进家庭,形成消费便捷、服务优良的现代服务网络体系。

六、深化重点领域改革,激发经济社会发展新活力

充分发挥市场在资源配置中的决定性作用,在重要领域和关键环节改革上聚焦发力,提高外向型经济发展水平。

深化经济领域改革。健全公司法人治理结构,推动企业股权多元化,加快建立现代企业制度。深化农村各项改革,以农村土地确权为重点,加快农村土地承包经营权流转,促进农村土地规模经营、集约经营,积极构建新型农业经营社会化服务体系。深化集体林权制度综合配套改革,完善林业双重管护、林权流转、抵押贷款等制度,大力发展林下经济。改进财政预算管理制度,做好“营改增”试点扩围工作。创新金融产品和服务,培育发展村镇银行、地方商业银行、小额贷款公司等新型金融机构,加大对实体经济、“三农”等重点领域的信贷投入。积极稳妥推进果品、设施蔬菜保险试点,扩大政策性农业保险范围。

加快行政体制改革。以优化机构设置、职能配置为目标,稳步推进市、县区政府机构改革。深化行政审批制度改革,逐步取消市场机制能够有效调节的经济活动审批。加快行政执法体制改革,稳步推进相对集中行政执法。进一步完善公共资源交易体制机制,建立统一规范有序的交易平台。市县(区)电子政务平台实现联网运行。推进事业单位分类改革,有序推动公办事业单位与主管部门理顺关系和去行政化,推进有条件的事业单位转为企业和社会组织。

推进社会事业改革。深化教育领域综合改革,创新教学管理、人才培养、考试招生制度和办学管理体制。继续推进医药卫生体制改革,巩固和扩大国家基本药物制度实施范围,落实乡村卫生服务一体化管理和一般

诊疗费制度,加快公立医院综合改革。坚持计划生育基本国策,启动实施“单独二孩”生育政策。深化文化体制改革,完善文艺院团法人治理结构。

不断扩大对外开放。深化与关中—天水经济区和陇东南城市的交流合作,联合申报、实施跨区域重大经济社会发展项目和丝绸之路世界文化遗产。坚持“走出去、引进来”,加大招商引资力度,力争实现到位引资265亿元,增长20%。积极参加区域合作组织活动和国内外重大节会活动,精心筹办甘肃省公祭伏羲大典暨第25届伏羲文化旅游节。筹建天水海关,加快国家级天水苹果产业外贸转型升级示范基地建设,力争全市外贸进出口增长10%。

七、协调发展社会事业,促进公共服务水平新提升

以实现发展成果更多更公平惠及全体人民为目的,努力为社会提供多样化服务,推动基本公共服务均等化。

优先发展教育事业。加快义务教育学校标准化建设,优化中小学布局结构,合理配置教育资源,加强素质教育,促进义务教育均衡发展。加快薄弱高中学校改造,加强职业教育基础能力及学科建设。全面落实义务教育经费保障机制、贫困学生资助和学生营养改善计划,强化学校安全工作,加强教师队伍建设,提高教育教学质量。加快市一中麦积校区(天水市渭滨中学)二期工程、市实验小学和市幼儿园新建项目、亚行贷款职业教育设施项目建设。加快发展学前教育,重视加强老年教育。

推动创新型城市建设。以“十大科技创新工程”为载体,组织实施国列、省列科技项目50项,促进科技创新、科技成果转化和科学普及等工作,积极争创国家级创新型试点城市。加快国家先进制造高新技术产业化基地建设,培育发展高新技术企业和民营科技企业,提升企业自主创新能力和核心竞争力。深化院地院企科技合作,推进科技特派员试点工作。加强知识产权保护,申请专利300件。推进“人才特区”试点建设,做好高层次和急需人才的引进培养和选拔使用工作。

加快文化体育事业发展。深入实施我市华夏文明传承创新区建设“136”工程,推进中华始祖文化园、大地湾国家级考古遗址公园、麦积山文化旅游区等项目规划与建设,加快甘谷大像山文化主题公园、清水轩辕文化产业园、张家川回乡风情园建设进度,培育壮大特色文化产业。健全公共文化体系,抓好天水文广传媒大厦、文化馆、图书馆、广电高山转播台站、地面数字电视无线覆盖等工程前期工作,加快市体育中心、市博物馆、天水大剧院和县区城市数字影院建设步伐。抓好麦积山石窟申遗、文化资源普查和文物保护工作,打磨提升地方特色文化剧目。大力开展群众文化活动,完成“千台大戏送农村”、农村电影放映工程年度任务。实施“体育惠民”工程,举办2014中国天水麦积山山地自行车邀请赛等活动。做好参加省第十三届全运会、第八届少数民族运动会等赛事组织工作。

提升公共卫生服务水平。抓好国家11项基本公共卫生项目和6项重大公共卫生服务项目实施,加快市妇幼保健院迁建,做好地方病监测防治工作,防止重大传染病和突发公共卫生事件发生。完善新农合管理制度,推进新农合医疗保障和医疗救助一站式服务。健全基层医疗卫生服务设施,提高群众保健养生水平,加强医德医风和医疗队伍建设,加快中医药事业发展。

八、着力保障改善民生,确保人民幸福指数新提高

以促进社会公平正义、增进人民福祉为根本出发点和落脚点,保障和改善民生,强化社会治理,确保社会既充满活力又和谐有序。

办好改善民生实事。在全面落实省上民生实事的同时,全力办好我市改善民生实事。(1)开工建设农村公路449条1351公里。(2)解决36万农村人口饮水安全问题。(3)实施整村推进项目120个。(4)硬化亮化城区小巷道100条40公里。(5)建成农贸市场10个、农家店

230个、便民蔬菜店50个。(6)新建改建农村乡镇中心幼儿园33所,实现全市乡镇中心幼儿园全覆盖。(7)提高新农合政府补助标准,落实27种重大疾病新农合保障。(8)建设标准化村卫生室50个。(9)通过各级各类招考扶持高校毕业生就业3000人。(10)城乡居民低保标准和补助水平分别提高15%。(11)完成就业培训3.95万人次、扶贫培训6万人次、农民科技培训12万人次。(12)实施保障性安居工程6606套54万平方米,改造农村危旧房12000户,为19770户住房困难家庭发放廉租补贴。(13)新建乡镇及社区体育健身中心8个,实施乡镇农民健身提升工程10个、村级农民健身工程100个。

努力扩大社会就业。全面落实各项扶持政策,完善就业创业服务和职业培训体系,促进以高校毕业生为重点的青年就业和农村转移劳动力、城镇困难人员、退役军人就业,力争年末高校毕业生就业率达到85%以上。规范公益性岗位开发管理,全力帮助各类困难人员就业。推进“素质增收”工程,抓好城乡居民职业技能培训。

完善社会保障体系。统筹做好社会保险、城乡低保、救灾救济、优抚安置等工作,不断扩大保障覆盖范围。落实被征地农民养老保险政策,做好村干部养老保险统筹工作。完善基本医疗保险市级统筹相关配套政策,稳步提高城镇基本医疗保险报销比例,重特大疾病医疗救助病种由7种扩大到26种。启动事业单位、社会团体参加工伤保险工作,年内参保率达到95%以上。重视老龄工作,推进农村互助老人幸福院建设工程,加快麦积全国综合养老示范基地等公益性养老服务设施建设。做好残疾人工作,加快市残疾人康复中心、县区残疾人托养服务中心和特殊教育学校建设。加快保障性住房建设,缓解低收入家庭住房问题。

全面加强社会治理。实施“六五”普法规划,推进依法治市进程。以社会治理“66158”工作计划为主线,深化“平安天水”建设。加强社会治安综合治理,完善立体化防控体系,加大网络监管力度,依法严厉打击各类违法犯罪活动。加强社会稳定风险评估和舆情分析研判,健全应急救援体系,提高突发事件处置能力。加强社区建设,全面推行网格化管理。落实信访工作责任制和领导包案制,及时办理群众通过书记市长留言板等渠道反映的合理诉求,建立涉法涉诉信访依法终结制度,加强人民调解和法律服务,做好社会矛盾排查和化解。全面落实安全生产“党政同责、一岗双责、齐抓共管”责任,深化重点行业领域安全隐患排查治理。切实加强市场监管和物价监测调控,强化食品药品专项整治。认真落实民族优惠政策,支持民族地区加快发展,推进张家川县“两个共同”示范县建设。依法管理宗教事务。重视保护妇女儿童合法权益。加强人民防空、民兵预备役建设和双拥共建工作,推动军民融合深度发展。认真做好统计、审计、外事侨务、地震、地方志和档案等工作。

九、加强政府自身建设,树立为民务实清廉新形象

以深化行政管理体制改革为主线,进一步转变政府职能,改进工作作风,提高行政效能,不断增强政府公信力和执行力。

着力转变政府职能。适应市场经济发展要求,积极推进政府职能由注重事前审批向市场监管、优化服务转变,对能够下放的权力一律下放,能够取消的收费一律取消,能够精简的审批一律精简,把工作重心放在优化发展环境、维护市场秩序、保障公平正义、提供公共服务等上来,进一步激发市场和社会主体的创造活力,增强经济发展的内生动力。

积极推进依法行政。坚持科学民主决策,不断完善公众参与、专家论证和政府决定相结合的决策机制,推动决策的科学化、民主化、制度化。加强政府法制和法治政府建设,坚持依法行政,积极争取将我市列入具有地方立法权的较大城市,自觉接受市人大、市政协以及各界群众和新闻舆论的广泛监督。推进政务、村务、厂务、校务公开,重视网络问政,坚持政府新闻发布和信息公开制度,促进政府工作公开公平公正、利民便民为民。

坚持改进工作作风。认真开展群众路线教育实践活动,自觉遵守中央八项规定和省、市委相关规定,坚决整治“形式主义、官僚主义、享乐主义、奢靡之风”,以良好的作风推动政府各项工作。政府部门和干部要把心思放到干事创业、推动发展上,少走形式、多干工作,少说空话、多办实事,少推诿扯皮、多解决问题,对工作既不能消极不干、也不能盲目蛮干、而要科学实干,倡导定了就干、干就干好的作风,减少文山会海,强化工作落实,加大效能问责力度,提高行政执行力,提升政府公信度。厉行勤俭节约,严格控制财政供养人员、公务接待、公务用车、办公用房,最大程度降低行政成本。

切实加强廉政建设。全面落实党风廉政建设责任制,切实抓好政府系统反腐倡廉工作。加强对重点领域、关键环节、重要岗位权力运行的监督制约,进一步规范经营性土地出让、建设工程招投标、矿产资源开发、政府采购等工作,强化行政监察和审计监督,严肃查处各类违纪违法案件。坚持廉洁从政,勤政为民,从政府领导班子成员做起,带头管好自己、管好亲属、管好身边工作人员,做到干部清正、政府清廉、政治清明。

各位代表,深化改革,加快发展,是全市人民的共同心愿,是各级政府的神圣职责。我们要在省委、省政府和市委的正确领导下,振奋精神,抢抓机遇,团结带领全市人民开拓创新,真抓实干,全面完成2014年经济社会发展各项任务,为把我市建设成为区域性现代化大城市而努力奋斗!
